# Catch and release patterns for target and bycatch species in the Northeast Atlantic deep-water shrimp fishery: Effect of using a sieve panel and a Nordmøre grid

**DOI:** 10.1371/journal.pone.0209621

**Published:** 2018-12-21

**Authors:** Roger B. Larsen, Bent Herrmann, Manu Sistiaga, Jesse Brinkhof, Juan Santos

**Affiliations:** 1 The Arctic University of Norway, UiT, Breivika, Tromsø, Norway; 2 SINTEF Ocean, Trondheim, Norway; 3 Thünen Institute of Baltic Sea Fisheries, Rostock, Germany; Department of Agriculture and Water Resources, AUSTRALIA

## Abstract

The Nordmøre grid and the sieve panel are two of the main devices used to reduce fish bycatch in trawl fisheries targeting shrimp species. However, even when using such devices, some small-sized fish enter the codend of the trawl together with the targeted shrimp. Therefore, bycatch reduction remains a problem in some shrimp fisheries. One such fishery is the Northeast Atlantic deep-water shrimp (*Pandalus borealis*) fishery where it is mandatory to use a Nordmøre grid. In this study, the bycatch reduction efficiencies and patterns for several fish species using the standard Nordmøre grid and an experimental sieve panel were investigated and compared. The effect of combining these devices was also explored. The bycatch reduction patterns differed significantly between the two devices and a more efficient bycatch reduction was obtained by combining them. However, while the loss of commercial-sized shrimp was only between 0 and 2% for the Nordmøre grid, it was between 37 and 56% for the tested sieve panel, making this completely unacceptable for commercial fishing. Therefore, before a sieve panel can be considered as an option for the fishery, other sieve panel designs that have a significantly lower loss of shrimp catches need to be identified.

## Introduction

The bycatch of juvenile fish in shrimp trawls is an issue that has been investigated worldwide [1, 2], including in the North Atlantic deep-water shrimp (*Pandalus borealis*) trawl fisheries. Work regarding bycatch reduction in the North Atlantic deep-water shrimp fishery in Norway started in 1970 [3] and has continued for almost five decades ([4–12]. In recent years, there has been an increased focus on sustainability and the fact that shrimp fisheries are often associated with high discard levels.

In certain fishing grounds and/or periods of the year, the density of juvenile fish caught with the targeted shrimp can be high. Thus, measures such as temporal area closures and the creation of protected areas are often implemented to minimize the potential environmental impact of shrimp fisheries [13]. Separating shrimp from juvenile fish is challenging, as the unwanted juvenile fish and the targeted shrimp are often quite similar in size. Therefore, any form of mechanical sorting such as sorting grids, will often not be effective. Apart from purely mechanical sorting, behavioral differences and differences in swimming capacity between the shrimp and juveniles of various fish species can potentially be used to separate shrimp from bycatch [14, 15]. However, most sorting devices are placed in the aft of the trawl, where fish juveniles normally arrive exhausted and have limited swimming capacity to escape.

**Fig 1:**
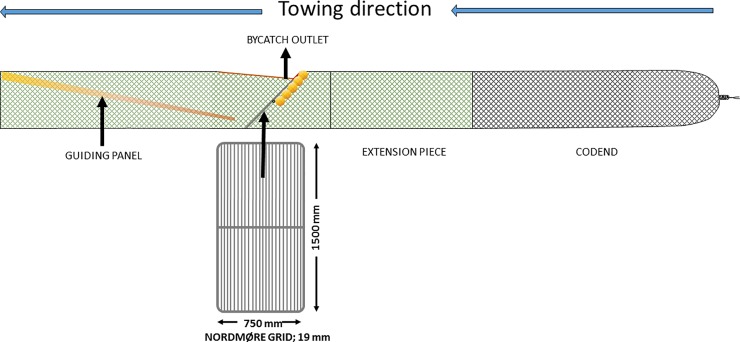
Illustration of a typical Nordmøre grid and codend design (side view).

**Fig 2:**
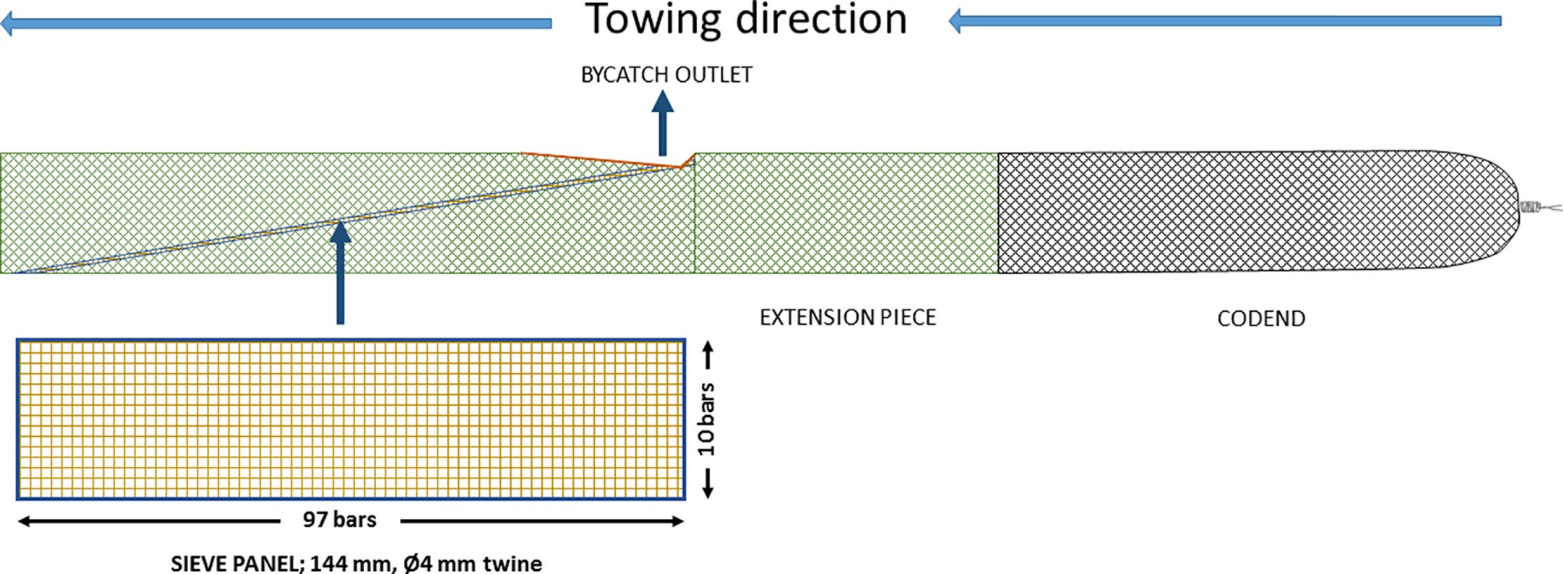
Illustration of a typical sieve panel and codend design (side view).

The Nordmøre grid (Fig 1) and sieve panels (also referred to as veil nets) or sieve nets (Fig 2) are the two most commonly used fish bycatch excluding devices in shrimp fisheries. The Nordmøre grid is mandatory in different shrimp fisheries in the US, Canada, Iceland and Norway [16], whereas sieve panels are mandatory in several of the European brown shrimp (*Crangon crangon*) fisheries including the Netherlands, United Kingdom, France, Germany, Denmark and Belgium [17, 18]. In all of these cases, the sorting device is used in combination with a size selective mesh codend. The Nordmøre grid system consists of a guiding panel, a steel or plastic grid mounted at an angle of 30–50°, and a triangular bycatch outlet in the upper panel just in front of the grid [12]. The individuals that pass between the bars of the Nordmøre grid continue towards the codend, while the others escape through the bycatch outlet. The working principle of a sieve panel is somewhat similar to that of the Nordmøre grid. A netting panel (normally a square mesh configuration) is installed in the extension piece ahead of the codend at an angle that typically varies between 10–30°. Thus, the individuals that pass through the mesh of the sieve panel continue towards the codend, whereas the individuals that do not are guided towards a bycatch outlet in the upper or lower panel of the section.

From 1984 to 1989, a 70 mm square mesh sorting panel (sieve panel) was compulsory during certain parts of the year in the Norwegian shrimp fishery. The panel was 4.1 m long and 2.6 m wide and it was installed at ca. 40^o^ in the aft of the trawl. During this period, several fishermen reported practical problems with the sieving properties of the panel. It was experimentally proven that large fish, especially flatfish, could accumulate on the panel surface, blocking the entrance to the codend. Shrimp loss was on average 10% but exceeded 15% by weight during certain periods [5]. In the late 1980s the possibility of examining other sieve panels, for example at a lower inclination angle, in Norwegian deep-water shrimp fisheries was not supported by industry and therefore not funded.

In Norwegian deep-water shrimp fisheries, the Nordmøre grid has proven to be an efficient juvenile fish bycatch excluding device [6, 9, 16]. However, there are still certain challenges associated with its use, in particular the risk of clogging the grid surface and low water flow behind the grid [19]. The low efficiency of sorting the smallest sizes of key bycatch species, for example redfish (*Sebastes* spp.), is also an important issue [16]. These and other challenges mean that in certain scenarios (e.g. areas with high densities of flatfish [20]) the grid underperforms, and alternative measures need to be investigated.

In the present study, a sieve panel was tested as an alternative to using the Nordmøre grid to mitigate bycatch of fish species in the Norwegian trawl fishery targeting deep-water shrimp. The possibility of using a sieve panel in front of the mandatory Nordmøre grid to further mitigate bycatch was also examined. It was hypothesized that a sieve panel with a low inclination angle installed in the extension piece would allow fish to escape more easily through the bycatch outlet without making physical contact, or at least not selective contact (see [21] for further information about the selectivity contact) with the sieve panel. In this manner, a low angle sieve panel may be better than a high angle sieve panel at guiding fish to the escape outlet. In addition, the ability of such a sieve panel to sort shrimp efficiently was examined. As the area available to make contact by shrimp is much larger on the sieve panel than the Nordmøre grid, a more efficient sorting process would be expected.

The trials presented in this study were designed to answer the following research questions:

- Does a sieve panel mounted at a low inclination angle represent a realistic alternative for bycatch mitigation in the Norwegian trawl fishery targeting deep-water shrimp?
- Would there be any benefit in terms of bycatch reduction in the Norwegian deep-water shrimp fishery by combining the use of a sieve panel and the mandatory Nordmøre grid?
- What would the loss of targeted deep-water shrimp be for a sieve panel compared to the Nordmøre grid, and is this loss dependent on the size of the shrimp?

## Materials and methods

### Ethics statement

The permit necessary to carry out the trials was obtained from the Directorate of Fisheries in Norway (permit number 16/12094) and none of the species sampled during the trials are endangered or protected. There were no additional ethical considerations linked to these experiments.

### Experimental design

The fishing trials were carried out onboard the Research Vessel (R/V) "Helmer Hanssen" (63.8 m Length Overall and 4,080 HP) between the 2^th^ and 4^th^ of March 2017 in the north of the Barents Sea (east of Hopen island, N76^o^00’–E32^o^00’).

A Campelen 1800# trawl built entirely of 80–40 mm diamond mesh (2 mm polyethylene twine) was used. The ground gear of the trawl was 19.2 m long and composed of three sections with 46 cm rubber discs. The doors used were of the type Thyborøn T2 (6.5 m^2^ and 2,200 kg). As the function of these doors changes with towing speed, depth and warp length, the door distance was kept constant at 48–52 m, independent of the towing depth by means of a 20 m long restrictor rope that was linked between the warps 80 m in front of the doors. The geometry of the trawl was monitored by a pair of Scanmar distance sensors and a Scanmar height sensor (Scanmar AS, Norway). The double sweeps between the doors and the trawl were 40 m long. The trawling time was kept constant at 45 ± 1 minutes.

The trawl was equipped with a four-panel grid section as illustrated in Fig 1. The grid section and transition sections had a mesh size of 50 mm (2 mm, polyamide). The grid, which is the standard grid used by the Norwegian inshore and coastal fleet targeting shrimp, was 1500 mm long and 750 mm wide. It was made of stainless steel, mounted so that it would be maintained at an angle of 45 ± 2.5° (mean ± SD) measured by a Scanmar grid sensor while fishing and had a bar spacing of 18.8 ± 0.4 mm. The escape opening on the top panel of the grid section was cut like a triangle equalling 35 meshes (cutting through 70 bars) in the N–direction (normal direction) and 70 meshes in the T–direction (transverse direction) as described in Isaksen et al. [6]. A sieve panel was mounted in the trawl section in front of the grid section as shown in Fig 2. The sieve panel was constructed of 143.73 ± 2.36 mm square mesh (4 mm polyethylene twine). The panel was 97 bars long and 10 bars wide and installed at an angle of ca. 9°. The escape opening in the top panel of the sieve panel section was cut with the same dimensions as for the grid escape opening. The mesh size in the sieve panel was measured by a wedge gauge and the bar spacing of the Nordmøre grid was measured using callipers following the guidelines described in Wileman et al. [22].

**Fig 3:**
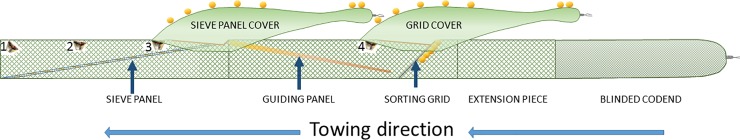
Illustration of the experimental gear design used during the trials (side view). The numbers 1, 2, 3 and 4 indicate the positions of underwater cameras during observations.

The fish and shrimp escaping from the openings of the sieve panel and the sorting grid were collected by covers made of 48 mm (2.1 mm polyethylene twine) and 35 mm (1.8 mm polyamide twine) diamond mesh. Both covers were made to be non-selective by adding small meshed netting, which had an average mesh size of 16.4 ± 0.5 mm. The codend had a 35 mm mesh size (2 mm polyamide twine), was blinded by a small meshed diamond netting (18.5 ± 0.5 mm) and was attached to the extension piece after the grid section (Fig 3).

The experimental gear used resulted in the catch being collected in three different compartments: i) the sieve panel cover, ii) the Nordmøre grid cover and iii) the blinded codend (Fig 3). The catch from the different compartments in the gear was kept separate at all times. The catch in each compartment was sorted by species and all the fish bycatch species were measured to the nearest centimeter below. No subsampling was carried out for any of the fish species, but the shrimp catch in all compartments for each haul was subsampled, as measuring all shrimp in the catch was practically impossible. A portion of the shrimp catch in each compartment weighing at least 1 kg was taken for each subsample. This was a random portion of the total catch which should be representative of the size distribution of the shrimp in that specific compartment. The carapace length of the shrimp was measured to the nearest millimeter below using callipers.

### Underwater recordings

To study shrimp and fish behavior with respect to the sieve panel, a GoPro Hero 4 Black Edition camera (San Mateo, California, USA) protected by a stainless-steel housing (iQsub Technologies, Czech Republic), and two red light emitting diode (LED) torches with batteries (Brinyte, DIV01C-V and type CREE XPE R5, Shenzhen Yeguang Technology Co., Ltd, China) was used. Red LEDs were selected as a previous study [23] showed that red lights affect fish behavior less than the more-traditionally used white lights. Recently, similar effects were found with red LED lights in a study on snow crab (*Chionoecetes opilio)* [24]. The camera and lights were attached to the upper panel in several positions over the sieve panel (Fig 3).

### Modeling and estimation of selection processes in the sorting system

The probability that a fish or shrimp will be retained in the codend (*r*_*codend*_(*l*): overall retention probability of the selection system) upon entering the experimental gear section depends on the probability that it passes through the sieve panel (*p*_*panel*_(*l*): passage probability through the sieve panel) toward the Nordmøre grid, and that it subsequently also passes through this grid into the codend (*p*_*grid*_(*l*): passage probability through the Nordmøre grid conditioned passage through the sieve panel). The size selection processes in the sorting section can be described by the following model:

$$\begin{array}{c} e_{panel}(l,\nu_{panel})=1.0-p_{panel}(l,\nu_{panel})\\ e_{grid}(l,\nu_{panel},\nu_{grid})=p_{panel}(l,\nu_{panel})\times \left(1.0-p_{grid}(l,\nu_{grid})\right)\\ r_{codend}(l,\nu_{panel},\nu_{grid})=p_{panel}(l,\nu_{panel})\times p_{grid}(l,\nu_{grid}) \end{array}$$ (1)

Where *e*_*panel*_(*l*,***ν***_*panel*_) is the probability for an individual fish of length *l*, or shrimp with carapace length *l*, that enters the combined size selection system to escape through the outlet in front of the sieve panel. ***ν***_*panel*_ is a vector of parameters for the parametric model used to describe the sieve panel passage probability. *e*_*grid*_(*l*,***ν***_*panel*_,***ν***_*grid*_) is the probability that an individual fish or shrimp that enters the combined size selection system will escape through the outlet in front of the Nordmøre grid. ***ν***_*grid*_ is a vector of parameters for the parametric model used to describe the Nordmøre grid passage probability (conditional to entering the grid zone).

As different species differ in morphology and behavior, model (1) needs to be applied separately for the deep-water shrimp and the different fish bycatch species. As this study was examining how the sieve panel and Nordmøre grid performed on average, the analysis was made for data summed over all hauls. Thus, expression (2) was minimized, which is equivalent to maximizing the likelihood for the observed data of the length dependent number of individuals measured as retained in the codend (*nc*_*l*_) versus collected in the sieve panel cover (*np*_*l*_) and in the Nordmøre grid cover (*ng*_*l*_).

$$-\sum_{i=1}^{m}\sum_{l}\left\{\frac{{np}_{il}}{{qp}_{i}}\times ln(e_{panel}(l,\nu_{panel}))+\frac{{ng}_{il}}{{qg}_{i}}\times ln(e_{grid}(l,\nu_{grid}))+\frac{{nc}_{il}}{{qc}_{i}}\times ln(r_{codend}(l,\nu_{panel},\nu_{grid}))\right\}$$ (2)

Where *qc*_*i*_, *qp*_*i*_ and *qg*_*i*_ represent the sampling factors for the fraction of individuals measured in the blinded codend, sieve panel cover and grid cover for each haul *i*, respectively. The sampling factors can take a value in the range 0.0 to 1.0 (1.0 if all individuals are length measured). The outer summation in (2) is over the hauls conducted and the inner summation is over the length classes in the data.

Before (2) can be applied with (1) to estimate *p*_*panel*_(*l*,***ν***_*panel*_) and *p*_*grid*_(*l*,***ν***_*grid*_) it must be decided which parametric models should be considered as candidates for these two processes. Larsen et al. [16] used a model with the following form to describe the size-dependent probability of a shrimp or fish passing through the Nordmøre grid:

$$\begin{array}{c} p(l,\nu )=C\times \left(1.0-rc(l,\nu c)\right)\\ where\\ \nu =\left(\begin{array}{c} C\\ \nu c \end{array}\right) \end{array}$$ (3)

The same model was also applied in this study to describe the size-dependent probability for bycatch fish species and for shrimp to pass through the sieve panel. Model (3) is applied independently for the sieve panel and the Nordmøre grid. In model (3) the probability of contacting the grid or sieve panel is modelled by the length-independent parameter *C*, which has a value in the range of 0.0 to 1.0. An estimated *C* value of 1.0 for a species means that every individual of that species contacts the sieve panel or grid in a way that provides them with a length-dependent probability of passing through the device. Where an individual fish or shrimp does not contact the sieve panel or grid, or is poorly oriented when making contact, it is reflected in the *C* value. For the fish or shrimp contacting the grid, the size selectivity function *rc*(*l*,***νc***) models the length-dependent probability of passing through the sieve panel or Nordmøre grid. The vector ***νc*** is the vector of parameters of this selectivity model. In the study by Larsen et al. [16] the standard *Logit* size selection model [22] for *rc*(*l*,***νc***) was applied. In this case ***νc*** contains two parameters: *L50* that denotes the length of the species with 50% probability of being prevented from passing through the device; and *SR* that describes the difference in length between individuals with a 75% and 25% probability, respectively, of being prevented from passing through the device. Further details on model (3) and the parameters of this model are provided in [16].

In this study, excepting the *Logit* model, three other S-shaped size selection models *Probit*, *Gompertz* and *Richard* [22] were also considered as candidates for *rc*(*l*,***νc***). In the case of the first two, the parameters are the same as for the *Logit* model, while the *Richard* model requires an additional parameter *1/δ* [22]. The simpler and more traditional models in [22] that do not explicitly consider the contact parameter were also considered as candidates. Technically this corresponds to fixing the contact parameter *C* to 1.0. The above considerations mean that in total eight different models were considered for *p*(*l*,***ν***) for the sieve panel and the Nordmøre grid independently. This lead to a total of 64 (8x8) different models to be considered separately for each species investigated in modelling and estimation of the size selection processes using Eq (1) in expression (2) for the combined selection system (Fig 3). Therefore, each combination of the eight candidate models for *p*(*l*,***ν***) for the sieve panel (denoted *p*_*panel*_(*l*,***ν***_*panel*_)) and Nordmøre (denoted *p*_*grid*_(*l*,***ν***_*grid*_)) process was fitted to the experimental data using expression (2). The combination of models resulting in the lowest Akaike information criterion (AIC) value [25] was chosen for each species separately to describe the size selection processes in the combined system.

The ability of the selected model to describe the experimental data sufficiently well was based on calculating the corresponding p-value. In the case of poor fit statistics (p-value < 0.05 and deviance >> degree of freedom), the residuals were inspected to determine whether the poor result was due to structural problems when modelling the experimental data (model (1)) or if it was due to over-dispersion in the data [22].

Once the models were selected for each species and the corresponding model parameters were estimated, *r*_*codend*_(*l*,***ν***_*panel*_,***ν***_*grid*_), *p*_*panel*_(*l*,***ν***_*panel*_) and *p*_*grid*_(*l*,***ν***_*grid*_) respectively can be used to quantify the combined size selection and standalone size selection for the sieve panel and Nordmøre grid.

Efron 95% percentile confidence bands [26] for the sieve panel passage probability, the grid passage probability curve and the combined codend entry probability, as well as for the parameters describing the processes, were obtained using a double bootstrap method implemented using the software tool SELNET [27] applied in the analysis. For each species analyzed, 1000 bootstrap repetitions were conducted to estimate the 95% confidence limits (Efron percentile) (see [16] for further details).

## Results

A total of eight hauls were carried out during the experimental period. The towing speed was kept at 3.0 ± 0.3 knots during all hauls. Underwater video recordings provided important information about the behaviour of the fish and the shrimps. Moreover, the video recordings confirmed that the technical setup for both the sieve panel and the Nordmøre grid sections functioned as intended. As far as we could interpret from these video recordings, the bycatch outlet kept its triangular shape and there was good clearance for fish to be able to pass into the sieve panel cover. As towing times were restricted to 45 minutes, the weight of fish in the sieve panel cover was kept low and did not affect the shape of the meshes in the sieve panel.

Of all the relevant bycatch species in the Northeast Atlantic deep-water shrimp fishery, cod (*Gadus morhua*), haddock (*Melanogrammus aeglefinus*), American plaice (*Hippoglossoides platessoides*) and redfish (*Sebastes* spp.) were captured in sufficient numbers to be included in the analyses (Table 1). Length measurements were taken for a total of 2802 redfish, 643 cod and 3492 haddock, 2184 American plaice and 3037 deep-water shrimp.

**Table 1:** Summary of the number of individuals length measured in catch data in each haul. *np*, *ng*, and *nc* denote the number of individuals measured from the sieve panel cover, the grid cover and the blinded codend, respectively. Values in parentheses are subsampling ratios in percentage (weight ratio), which are provided only if subsampling took place.

	Redfish			Cod			Haddock			American plaice			Deep-water shrimp		
Haul	*np*	*ng*	*nc*	*np*	*ng*	*nc*	*np*	*ng*	*Nc*	*Np*	*ng*	*nc*	*np*	*ng*	*nc*
1	93	40	32	38	4	5	24	3	35	200	9	83	159(5.0)	6	188(4.1)
2	117	67	7	64	9	2	41	1	4	206	28	56	163(9.6)	13(86.7)	175(4.5)
3	42	8	10	15	0	0	66	0	18	211	5	90	164(20.8)	9	196(11.6)
4	72	8	15	13	0	4	110	2	19	207	16	55	151(14.4)	5	240(8.2)
5	94	4	35	22	0	1	207	0	15	264	6	59	175(7.4)	3	252(7.7)
6	545	45	101	97	3	10	1097	2	67	212	6	25	191(3.9)	14	227(8.6)
7	156	195	303	63	63	37	353	176	393	86	21	58	137(21.5)	6	216(4.2)
8	416	36	361	130	16	47	583	19	257	225	5	51	148(6.6)	0	199(3.5)
sum	1535	403	864	442	95	106	2481	203	808	1611	96	477	1288	56	1693

From the 64 models tested, the model with the lowest AIC value was chosen to represent the data for each species. For redfish, the *Richard* model with *C*_*grid*_ fixed at 1.0 described the data for the sieve panel passage process best, whereas the *Probit* with an estimated *C*_*grid*_ resulted in the lowest AIC value for the Nordmøre grid passage process (Table 2).

**Table 2:** Parameter values and fit statistics for the selected models. Values in parentheses are 95% confidence limits. Note that *L50* and *SR* are provided in cm (total length) for the fish bycatch species and in mm (carapace length) for deep-water shrimp. * not in model.

	Redfish	Cod	Haddock	American plaice	Deep-water shrimp
Panel model	*Richard*	*Gompertz*	*Logit*	*Probit*	*Gompertz*
Grid model	*Probit*	*Probit*	*Richard*	*Gompertz*	*Gompertz*
*C*_*panel*_ (%)	Fixed at 100	Fixed at 100	Fixed at 100	Fixed at 100	Fixed at 100
*L50*_*panel*_	13.8 (0.1–22.6)	4.4 (0.1–23.5)	3.3 (0.1–16.5)	11.9 (10.4–14.0)	27.3 (20.9–49.6)
*SR*_*panel*_	30.3 (12.8–53.6)	49.1 (18.8–100.0)	30.3 (12.7–57.0)	13.4 (9.3–13.3)	30.8 (15.3–100.0)
*1/δ*_*panel*_	0.004 (0.002–100.0)	*	*	*	*
*C*_*grid*_ (%)	99.3 (96.0–100.0)	96.9 (90.2–100.0)	91.6 (78.2–100.0)	99.4 (98.0–100.0)	100.0 (99.9–100.0)
*L50*_*grid*_	15.4 (14.7–16.3)	22.4 (21.4–24.5)	19.7 (18.5–21.4)	19.5 (17.9–22.0)	56.4 (42.9–88.2)
*SR*_*grid*_	2.3 (0.3–2.9)	2.3 (0.1–3.1)	2.8 (0.1–4.4)	7.6 (5.3–10.7)	24.5 (14.1–49.2)
*1/δ*_*grid*_	*	*	0.018 (0.010–100.0)	*	*
P-value	0.139	0.392	0.900	0.998	< 0.001
Deviance	91.63	83.86	23.96	50.87	95.53
DOF	78	81	34	83	37

Therefore, this combination of models was chosen for redfish (Table 2). Regarding cod, the *Gompertz* model with *C*_*grid*_ fixed at 1.0 and *Probit* model with an estimated *C*_*grid*_ resulted in the models with the lowest AIC value for the sieve panel process and Nordmøre grid process, respectively. For haddock, the *Logit* model with *C*_*grid*_ fixed at 1.0 described the data for the sieve panel passage process best, whereas the *Richard* model with an estimated *C*_*grid*_ resulted in the lowest AIC value for the Nordmøre grid passage process. For American plaice, the *Probit* model with *C*_*grid*_ fixed at 1.0 described the data for the sieve panel passage process best, whereas the *Gompertz* model with an estimated *C*_*grid*_ resulted in the lowest AIC value for the Nordmøre grid passage process. The *Gompertz* model with *C*_*grid*_ fixed and an estimated *C*_*grid*_ described the deep-water shrimp data best for the sieve panel and the Nordmøre grid, respectively.

**Fig 4:**
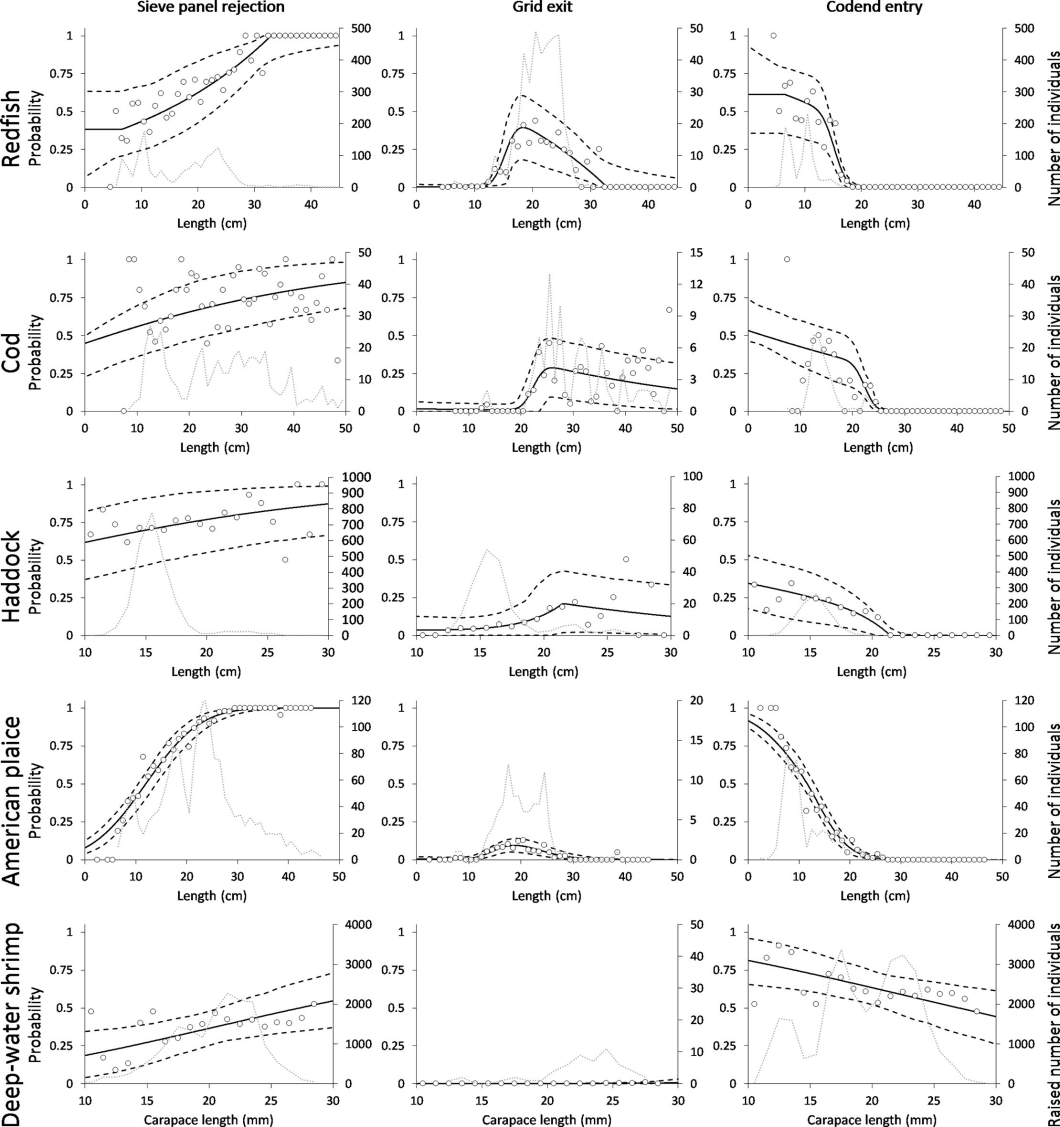
Modelling of the length-dependent observed experimental release and retention rates. Left column: release share to sieve panel cover (sieve panel rejection probability). Centre column: release share to Nordmøre grid cover (grid exit probability). Right column: catch share to blinded codend (codend entry probability). Data for redfish are shown in the first row, followed by cod, haddock, American plaice and deep-water shrimp in the bottom row. Circle marks represent the observed experimental rate and the solid black curve represents the modelled rate. Black dashed curves represent 95% confidence bands for the modelled rate and the grey dotted curve represents the observed experimental catch in the specific compartment (sieve panel cover, grid cover and blinded codend).

The fit statistics show that the chosen models represent the data well for all bycatch species (Table 2). All p-values obtained were >0.05, which means that the discrepancy between the data and the modelled length dependent curves for the catch in the sieve panel cover, the grid cover and in the codend can be coincidental. This is corroborated by comparing the deviance and degrees of freedom, which for all bycatch species was of the same magnitude. For the shrimp, the estimated p-value was <0.05. However, the modelled catch share curves follow the main trend in the experimental data (Fig 4). Therefore, it was assumed that the low p-value obtained was a consequence of over-dispersion in the experimental data that resulted from working with pooled and subsampled data with low sampling rates (Table 1). Such cases have previously led to low p-values and high dispersion [28–30]. The values for the selectivity parameters obtained from the analysis, and their application to describe the length-dependent device passage probability (size selectivity) for the sieve panel and Nordmøre grid, are presented in Table 2. It should be noted that the *L50*_*grid*_ and *SR*_*grid*_ values obtained for shrimp were far above any biological size range and should be interpreted as parameter values that make the model describe the grid passage probability for the sizes of shrimp available. In general, both the *C*_*panel*_ and the *C*_*grid*_ values were very high (close to 100%), demonstrating that nearly all individuals of fish and shrimp that enter the zone of each of the selective devices contact them and will be subjected to a size selective process. Furthermore, for all cases the 95% confidence bands for *C*_*grid*_ include the 100% contact value, which demonstrated that based on the collected data there was no significant difference from the 100% contact value in any of the cases.

**Fig 5:**
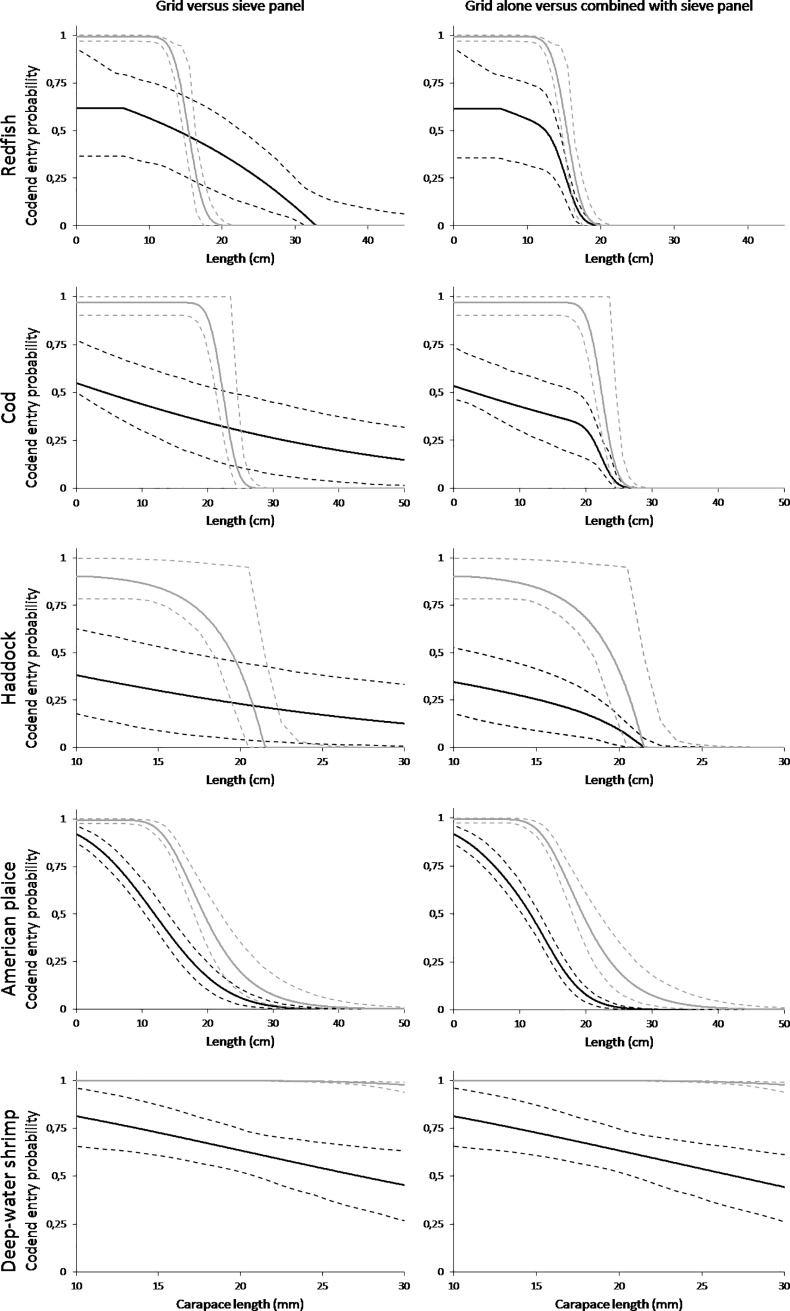
Predicted length-dependent codend entry probabilities. Left column shows the Nordmøre grid standalone deployment (grey curve) versus the sieve panel standalone deployment (black curve). The right column shows the Nordmøre grid standalone deployment (grey curve) versus the Nordmøre grid and combined sieve panel (black curve). Graphs in the top row show data for redfish, followed by cod, haddock, American plaice and deep-water shrimp in the bottom row. Solid curves represent the predicted probabilities. Dashed curves represent 95% confidence bands for the predicted probabilities.

The results show that the sieve panel rejects a large fraction of the smallest redfish, haddock and cod resulting in far fewer individuals reaching the Nordmøre grid when the sieve panel is used. For American plaice, only a few of the smallest individuals were rejected by the sieve panel. However, the rejection rate increases quickly with increasing fish size, meaning that overall fewer American plaice will reach the grid zone. The insertion of the sieve panel and the resulting rejection rates led to significantly lower codend entry rates for the bycatch species than with the Nordmøre grid alone. Based on the modelled catch sharing rates (Fig 4, Table 2) and using model (1), estimates for the length-dependent codend entry probability were obtained for the Nordmøre grid and sieve panel individually (Fig 5 left column), and in combination (Fig 5 right column).

In general, the pattern for the length-dependent codend entry probability differs significantly between the Nordmøre grid and the sieve panel when deployed alone (Fig 5 left column). Overall, for round fish species (redfish, cod and haddock) the sieve panel showed a significantly lower codend entry probability for the smaller sizes of fish, but higher entry probabilities for bigger sizes compared to the Nordmøre grid alone. Therefore, the sieve panel was more efficient at avoiding codend entry of the smallest round fish, but less effective for avoiding codend entry of the bigger sizes. For American plaice, the only flatfish species in the dataset, the sieve panel was found to be more effective at preventing fish entry to the codend.

Using the selectivity of the Nordmøre grid as a baseline, the increase of bycatch reduction by adding the sieve panel in front of the mandatory Nordmøre grid was quantified. In general, for the bycatch species investigated, adding a sieve panel in this position significantly reduced the entry probability of fish to the codend (Fig 5 right column). Based on these results, the first impression could be that while replacing the Nordmøre grid with a sieve panel would not work, it would be advantageous to mount it in front of the Nordmøre grid to complement the selection of the grid. However, the consequences on the catch efficiency of the targeted deep-water shrimp also needs to be considered, and regarding this, the tested design of the sieve panel showed a negative effect (Fig 5 bottom row). In general, the codend entry probability was much lower with the sieve panel than with the Nordmøre grid, and this difference increases with increasing shrimp carapace length. For a shrimp with a carapace length of 20 mm, the codend entry probabilities were 63% and > 99% with the sieve panel and the Nordmøre grid, respectively, while these probabilities were 44% and 98% at 30 mm carapace length. When the sieve panel and the Nordmøre grid were combined, the entry probabilities were almost the same as for the sieve panel alone because the entry probability through the Nordmøre grid was high. Thus, our results demonstrate that the passage probability for shrimp through the sieve panel needs to be improved considerably before this system could potentially be used in the commercial deep-water shrimp fishery of the Northeast Atlantic. In particular, the loss of the biggest and most valuable shrimp using the tested design is unacceptable for the industry.

## Discussion

Since the early 1990s, bycatch mitigation in the deep-water shrimp fisheries of the Northeast Atlantic have almost exclusively been carried out by means of the Nordmøre sorting grid and size selective codends [6, 16]. However, in different European shrimp fisheries, apart from the more widespread sorting grids [8, 31], sieve panels and other soft panel excluders have also been shown to be effective at sorting bycatch without major losses of brown shrimp [18]. One could argue that the bycatch sorting capacity of the selectivity devices is the most important characteristic for these types of devices, but another crucial aspect to consider is the loss of the targeted shrimp created by the installation of such a device. Four different designs of sieve nets constructed with different mesh sizes were tested in a Crangon fishery [32]. In general, the results showed that the sieve nets tested were effective at reducing the bycatch of important commercial species, while the loss of shrimp was estimated at 12% or less. Similarly, in an experiment carried out in the North Sea brown shrimp fishery, good bycatch sorting properties and a loss of commercial brown shrimp of around 15% or less when using sieve nets were reported [18]. Furthermore, in a social, economic and biological analysis of the brown shrimp fishery in the UK, it was estimated that the use of sieve nets leads to an average juvenile fish bycatch reduction of 36 ± 4% and a shrimp loss of 14 ± 3% [33]. In general, sieve panels and nets show good bycatch sorting properties, but sorting grids seem to have lower shrimp loss percentages. For example, Graham [31] reported losses of ca. 10% with different grid designs in the brown shrimp fishery, while Grimaldo and Larsen [9] reported losses of ca. 5% in the Barents Sea deep-water shrimp fishery using a Nordmøre grid. However, when given the choice, sieve panels and nets are often preferred by fishermen as they involve fewer handling challenges and the risk for blockage is smaller than with sorting grids [17].

Earlier studies from the brown shrimp fishery and other fisheries around the world have shown that sieve panels or similar devices can be an effective way to release bycatch from trawls without major shrimp losses [18, 32, 34–36]. Thus, the aim of the present study was to evaluate whether such a sieve panel could be a realistic alternative to sorting grids in the deep-water shrimp fishery, or if installing such a device in addition to the Nordmøre grid could improve the bycatch sorting process for this fishery in any way. The results showed that the sieve panel tested can complement the Nordmøre grid at reducing the bycatch of juvenile fish species significantly. However, the tested version of the sieve panel led to a length dependent shrimp loss of 37% and 56% (by number) for carapace lengths of 20 and 30 mm, respectively. For all bycatch species included in this study, adding a sieve panel in front of the Nordmøre grid improved the release of juvenile fish significantly. However, based on the results obtained, the main conclusion of this study is that the tested sieve panel design cannot be considered as an alternative to the Nordmøre grid in its present form.

**Fig 6:**
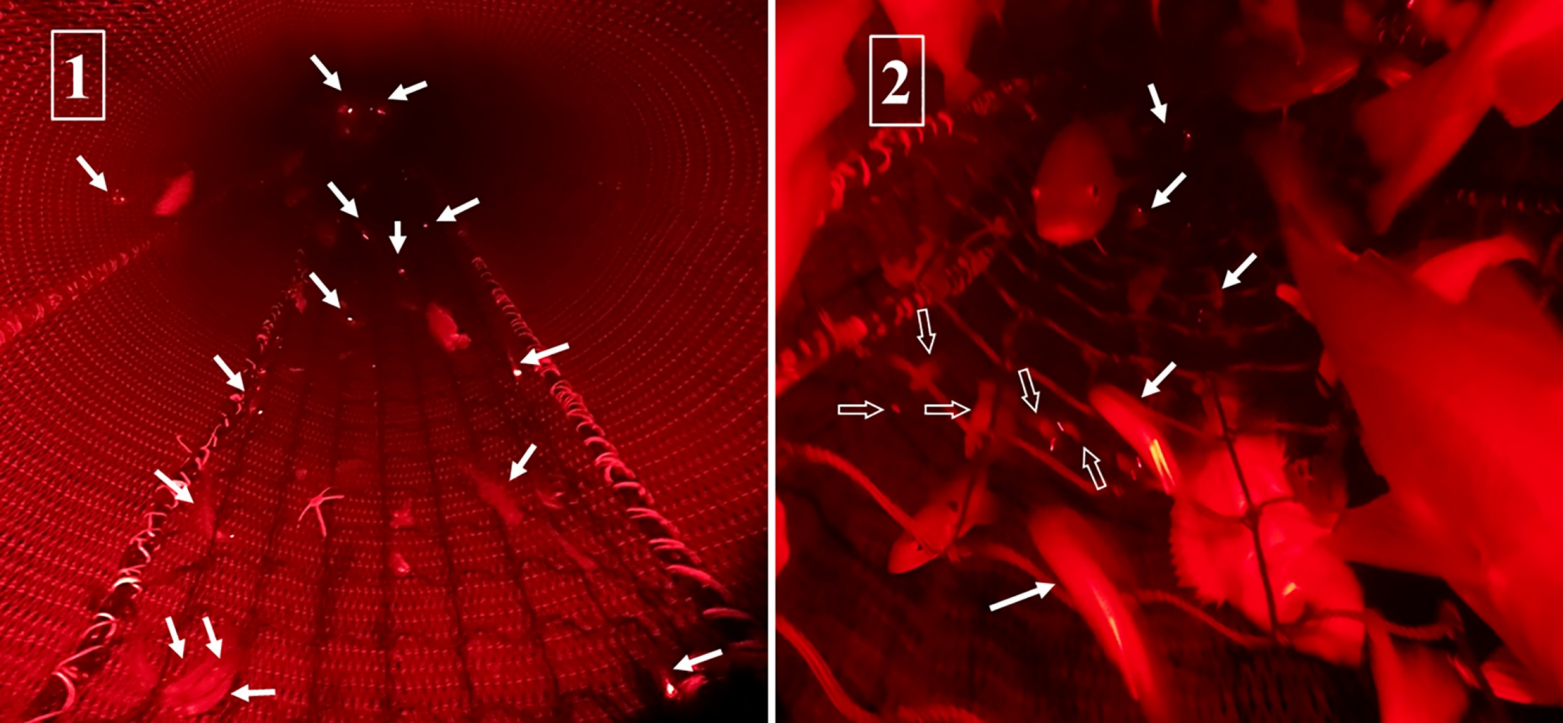
Photos taken from underwater recordings made during March 2017. Plate 1 shows the start (front) of the sieve panel. Solid white arrows indicate shrimp over the sieve panel. Plate 2 shows the aft part of the sieve panel close to the bycatch outlet. Hollow arrows indicate shrimp under the sieve panel.

The sieving ability of a veil net or sorting panel is related to mesh size and its orientation and the operating angle. At very low operating angles, the projected vertical distance between bars of a square mesh panel will be small. With a 144 mm sieve panel installed at angle of 9^o^, the projected vertical distance between 72 mm long bars equals 11.3 mm (72×*sin*(9)). Despite the sieve panel being 7 m long (i.e. 97 bars), it was able to guide a large fraction of small fish and shrimp to the bycatch outlet. Underwater recordings revealed that many shrimp and fish easily bounced off the panel and slid from bar to bar all the way to the bycatch outlet (Fig 6). This effect also explains why [5] found a relatively small shrimp loss (on average ca. 10% by weight) with 70 mm square mesh panels. On the other hand, it was clear that the rather steep (i.e. ca. 40^o^) sorting panel led to poor exclusion of fish below 25 cm and in areas with large quantities of fish, the panel would easily clog leading to a dramatic increase in shrimp exclusion.

The results of this study, as in many other cases, show the importance of thoroughly studying the properties of a new selection device before it is commercially applied in a fishery. Sieve panels have been shown to work fairly well in several fisheries around the world, but applying the design tested in this study in its current form in the deep-water shrimp fishery would have led to serious negative consequences for the effectiveness of the fishery. It remains to be seen if alternative sieve panel designs can be at least as effective as the panel tested in this study at releasing fish bycatch without significantly affecting shrimp catches. The effect of increased sieve panel mesh size and inclination angle could potentially increase the shrimp passage probability, but such designs may also lead to higher passage probabilities for bycatch species. Based on the results from this study, new experimental trials that investigate the performance of other sieve panel designs with different mesh sizes and operational angles are recommended.
